# Performance of two rapid point of care SARS-COV-2 antibody assays against laboratory-based automated chemiluminescent immunoassays for SARS-COV-2 IG-G, IG-M and total antibodies

**DOI:** 10.1016/j.plabm.2021.e00201

**Published:** 2021-01-19

**Authors:** C.S. Lau, S.P. Hoo, Y.L. Liang, S.K. Phua, T.C. Aw

**Affiliations:** a Department of Laboratory Medicine, Changi General Hospital, Singapore; b Department of Medicine, National University of Singapore, Singapore; c Academic Pathology Program, Duke-NUS Medical School, Singapore

**Keywords:** SARS-CoV-2, Antibodies, Assay evaluation, Point-of-care testing

## Abstract

**Introduction:**

We evaluated two SARS-CoV-2 antibody point-of-care tests (POCTs) (Abbott Panbio COVID-19 IgG/IgM and Roche SARS-CoV-2 Rapid Antibody tests) and compared the results to their respective chemiluminescent immunoassays (CLIAs) (Abbott Architect IgM, Architect IgG, Roche Cobas total antibody assays).

**Method:**

200 pre-pandemic sera and 48 samples positive for various conditions (18 viral hepatitis, 18 dengue, 11 ANA and 1 dsDNA) were used as controls and to assess for cross-reactivity. Anonymised residual leftover sera positive for SARS-CoV-2 on RT-PCR were recruited as cases (n ​= ​133). The sensitivity/specificity/cross-reactivity/positive predictive value (PPV)/negative predictive value (NPV) of the POCTs were assessed. Concordance between the POCTs and chemiluminescent immunoassays (CLIAs) were analysed.

**Results:**

Abbott/Roche POCT specificity was 98.7%/100% (95% CI 96.5–99.8/98.5–100) and sensitivity was 97.2%/97.2% (95% CI 85.5–99.9/85.5–99.9) in cases ≥14 days post-first positive RT-PCR (POS), PPV was 68.7%/100% (95% CI 41.3–87.2/94.7–100.0), and NPV was 97.4%/97.6% (95% CI 97.0–97.8/97.2–98.0). In cases ≥14 days POS, concordance of Abbott/Roche POCT and CLIAs was 97.2%/100% (35/36 and 36/36 results). The sensitivity of individual IgM-band results on both POCTs did not increase >95% even after 14 days POS (Abbott 2.78%, Roche 44.4%).

**Conclusion:**

Both POCTs have good specificity, little cross-reactivity with other antibodies, and sensitivity >95% when used in subjects ≥14 days POS. Analysis of individual POCT IgG/IgM-bands did not provide any additional information. POCTs can substitute for CLIAs in cases ≥14 days POS. In low prevalence areas, POCTs would be especially useful when combined with antigen testing in an orthogonal format to increase the PPV of COVID-19 results.

## Highlights

• The POCT IgM-band is negative in most cases of COVID-19.
• There is little utility in examining the IgG-IgM bands individually.
• Like CLIAs, the sensitivity of the POCTs is >95% 14 days after RT-PCR positivity.
• Combining POCTs with another test orthogonally improves the PPV.

## Abbreviations

SARS-CoV-2: Novel severe acute respiratory syndrome coronavirus 2
COVID-19: Coronavirus disease 2019
RT-PCR: Real-time polymerase chain reaction
CLIA: chemiluminescent immunoassays
ELISA: enzyme-linked immunosorbent assays
LFIA: lateral flow immunoassays
POS: Post-first positive RT-PCR
POCT: point-of-care tests
HS: health screening
ANA: anti-nuclear antibody
ds-DNA: double-stranded DNA antibody
COI: Cut-off index
PPA: Positive percentage agreement
NPA: Negative percentage agreement
PPV: Positive predictive value
NPV: Negative predictive value

## 1 Introduction

Current novel severe acute respiratory syndrome coronavirus 2 (SARS-CoV-2) antibody immunoassays are mostly qualitative and include chemiluminescent immunoassays (CLIAs), enzyme-linked immunosorbent assays (ELISA), and lateral flow immunoassays (LFIA). We have previously evaluated the performance of CLIAs from Abbott [1] and Roche [2] and found them excellent. However, automated CLIAs require samples to be delivered to a central laboratory for analyses. Arguments have been made in support of the use of point-of-care tests (POCT) for SARS-CoV-2 antibodies (typically LFIAs) because of immediacy and convenience of results. Although the initial experience with POCT assays before April 2020 were disappointing [3], newer POCT assays have recently emerged. The US Food and Drug Administration has included 56 antibody tests under its Emergency Use Authorizations, with 15 out of 16 LFIAs approved after April 2020 [4]. There is little information on how the newer generation of LFIAs compare to CLIAs. We thus evaluated the performance of the newly released Abbott Panbio COVID-19 IgG/IgM Rapid Test and the Roche SARS-CoV-2 Rapid Antibody test and compared both POCTs to the Abbott Architect IgG and Architect IgM CLIAs as well as Cobas total antibody CLIA in SARS-CoV-2 reverse transcriptase polymerase chain reaction (RT-PCR) positive subjects and Coronavirus disease 2019 (COVID-19) naive cases.

## 2 Methods

### 2.1 Study subjects

Residual leftover sera were used in this study. Two hundred pre-pandemic samples from a staff health screening (HS) program in 2018 served as controls. In addition, a panel of 48 antibody positive sera (18 hepatitis B/C/E, 18 dengue, 11 anti-nuclear antibody [ANA] and 1 double-stranded-DNA antibody [dsDNA]) were used to assess for potential cross-reactivity. All control and potential confounding test samples (N ​= ​248) were non-reactive on the Architect IgG and Architect IgM assays and were deemed to be free of COVID-19. De-identified residual sera from other routine laboratory testing (e.g. renal panels, blood cell counts) from subjects who tested positive for SARS-CoV-2 on RT-PCR from April to June 2020 were recruited as cases (N ​= ​133). Days POS was used as a surrogate for disease onset, and results were stratified according to days POS. The mean age of the RT-PCR positive cases was 51.0 ​± ​17.7years and the mean age of the controls was 47.2 ​± ​12.7years. The male/female distribution of the cases was 81.2%/18.8% (108/25) and 20.9%/79.1% (49/185) for the controls. No subjects underwent repeated testing on any assay.

### 2.2 Materials and methods

The Abbott Panbio COVID-19 IgG/IgM Rapid Test device is a qualitative immunochromatographic SARS-CoV-2 IgG and IgM LFIA. Serum/plasma (10μL) is applied into the specimen well with two drops (approximately 60 ​μL) of buffer. The mixture migrates along a membrane strip, where they interact with anti-human IgG and anti-human IgM antibodies to create a visible result. A visible control line (precoated with goat anti-rabbit antibodies) indicates that the result is valid. The test is considered positive when the control, IgG and/or IgM test lines are all visible. The Roche SARS-CoV-2 Rapid Antibody (POCT) test is also a LFIA that uses a similar principle. Monocolonal chicken antibodies (conjugated with colloidal gold particles) coat a control line, monoclonal anti-human IgG/IgM antibodies coat their respective lines. SARS-CoV-2 specific antibodies in the sample first react with the control line to form complexes and then reach the M and G test lines to produce a visible result. For both POCTs, a sample is considered as reactive if either IgG and/or IgM bands are positive. Both assays require a 10-min reaction time followed by a read time between 10 and 20 ​min (Abbott) and 10–15 ​min (Roche); results beyond 20 ​min were rejected.

For RT-PCR testing, our hospital molecular laboratory employs a duplex real-time RT-PCR that targets the N and E genes using a Qiagen EZ1 extraction system and Rotor Gene Q amplification system. The Abbott Architect SARS-CoV-2 IgG assay is a qualitative CLIA run on the ARCHITECT i2000 System, as described previously [1]. The stated cut-off index (COI) of the Architect IgG assay is 1.4. In our hands [1], the Architect IgG assay had a CV of 3.4% (COI ​= ​0.06) and 1.6% (COI ​= ​8.6); assay specificity was 99.8%, and sensitivity of 96.7% for samples ≥14 days POS and 45.9% for samples <7 days. The Architect SARS-CoV-2 IgM antibody assay is also a CLIA that uses a similar principle to the Architect IgG assay, resulting in a signal (relative light unit) that is directly proportional to the amount of SARS-CoV-2 IgM titres and reflected in a COI; a COI of >1.0 is regarded as reactive. The manufacturer reported precision of the assay is 2.8/2.9% (COI of 1.96/2.80), with a reported sensitivity of 59.5% (0–7 days POS) to 100% (>14 days POS) and a specificity of 99.7%. The Roche SARS-CoV-2 total antibody assay is also a CLIA and has been described previously [2]. It has a stated COI of 1.0, and in our hands, CV was 2.9% (COI ​= ​0.1) and 5.1% (COI ​= ​3.0), specificity was 99.9%, and sensitivity was 97.1% for samples ≥14 days POS.

All tests were performed by the five authors who are all laboratory trained. All serum samples were stored at 4 ​°C, −20 ​°C or −70 ​°C. Reagents were deployed according to the manufacturer’s requirements: for POCTs at room temperature, and for CLIAs reagents were stored at 4 ​°C until used.

### 2.3 Statistical analysis

As the tests involved are all qualitative tests, the diagnostic specificity is represented by the negative percentage agreement (NPA) between antibody negativity against all control subjects; diagnostic sensitivity is represented by the positive percentage agreement (PPA) between antibody positivity against all RT-PCR positive patients. The positive predictive value (PPV)/negative predictive value (NPV)/cross-reactivity of the assays were assessed. We also explored concordance between the Abbott POCT/combined Architect IgG and IgM results as well as the Roche POCT/Cobas antibody results in cases and controls. No data with indeterminate or missing results was used. Data were presented in either mean ​± ​standard deviation or median [inter-quartile range], as appropriate. 95% confidence intervals for sensitivity and specificity were calculated according to Clopper and Pearson (“exact” method) with standard logit confidence intervals for predictive values. Paired categorical data were compared using the McNemar test and a p-value <0.05 was considered as statistically significant. Statistical analyses were performed using MedCalc® Statistical Software version 19.5.3 (MedCalc Software Ltd, Ostend, Belgium). For the 95% confidence interval in groups with 100% PPV we used Stata 14 software (StataCorp. 2015. Stata Statistical Software: Release 14. College Station, TX: StataCorp LP). Our IRB deemed this work exempt as this was part of routine laboratory evaluation of new assays and seroprevalence surveillance using de-identified leftover sera. Compliance with STARD guidelines is enclosed (see Supplemental Table 1).

## 3 Results

### 3.1 Cross-reactivity, specificity and concordance of controls

One subject positive for hepatitis B, and 1 ANA positive subject had positive IgM-bands on the Abbott POCT only (see Table 1); both of these samples were non-reactive on the Architect IgG and IgM assays (COI <0.03 for both). All dengue cases (n ​= ​18) were not reactive on both POCT assays. Out of 248 non-reactive controls, two cross-reactivity samples were positive on the IgM-band, and one pre-pandemic HS sample was IgG positive on the Abbott POCT, and none were reactive on the Roche POCT (see Table 1). This yielded a specificity of 98.7% (95% CI 96.5 to 99.8) on the Abbott POCT, and a specificity of 100% (95% CI 98.5 to 100) on the Roche POCT. Concordance between the Abbott POCT and Abbott CLIA was 98.8%, and concordance between the Roche POCT and Roche CLIA was 100%

**Table 1:** Specificity and cross-reactivity of the Abbott and Roche POCT assays.

Group	N	Abbott Rapid IgG	Abbott Rapid IgM	Roche Rapid IgG	Roche Rapid IgM
Hepatitis	18	0	1^a^	0	0
Dengue	18	0	0	0	0
ANA	11	0	^1^^b^	^0^	^0^
dsDNA	1	0	^0^	^0^	^0^
Pre-pandemic	200	1^c^	0	0	0
Total	248	1	2	0	0

Abbreviations: POCT: point-of-care test, COI: cut-off index, ANA: anti-nuclear antibody, dsDNA: double-stranded-DNA antibody.

a COI of this patient on the Architect CLIA: IgG 0.03, IgM 0.02.

b COI of this patient on the Architect CLIA: IgG 0.02, IgM 0.03.

c COI of this patient on the Architect CLIA: IgG 0.02, IgM 0.04.

### 3.2 Sensitivity and concordance of cases

We analysed the PPA (sensitivity) of the assay in 133 SARS-CoV-2 RT-PCR positive patients. When we compared the sensitivities of all four assays, all assays had sensitivity <50% within the first week of infection. However, performance subsequently improved to >95% for all assays after 14 days POS (see Table 2, Table 3). The POCT assays agreed closely with the CLIA results after 14 days POS (Abbott 97.2% concordance, Roche 100% concordance).

**Table 2:** Sensitivity analysis by days post first positive RT-PCR of Abbott POCT vs CLIA.

Days POS	N	Abbott POCT		Abbott IgM ​+ ​IgG		Concordance	
		Pos/Neg	PPA (95% CI)	Pos/Neg	PPA (95% CI)	Yes/No	Percentage concordance
0 to 6	65	7/58	10.8 (4.44–20.9)	20/45	30.8 (19.9–43.4)	52/13	80.0
7 to 13	32	25/7	78.1 (60.0–90.7)	25/7	78.1 (60.0–90.7)	32/0	100
≥14	36	35/1	97.2 (85.47–99.9)	36/0	100 (90.3–100)	35/1	97.2

Abbreviations: POS: post-first positive RT-PCR, POCT: point-of-care test, Pos: positive, Neg: negative, PPA: positive percentage agreement.

**Table 3:** Sensitivity analysis by days post first positive RT-PCR of Roche POCT vs CLIA.

Days POS	N	Roche POCT		Roche total antibody		Concordance	
		Pos/Neg	PPA (95% CI)	Pos/Neg	PPA (95% CI)	Yes/No	Percentage concordance
0 to 6	65	12/53	18.5 (9.92–30.0)	10/55	15.4 (7.63–26.5)	51/14	78.5
7 to 13	32	25/7	78.1 (60.0–90.7)	25/7	78.1 (60.0–90.7)	32/0	100
≥14	36	35/1	97.2 (85.5–99.9)	35/1	97.2 (85.5–99.9)	36/0	100

Abbreviations: POS: post-first positive RT-PCR, POCT: point-of-care test, Pos: positive, Neg: negative, PPA: positive percentage agreement.

As there appeared to be difference in the results from 0 to 6 days POS, we performed McNemar testing between the POCT and CLIA in these cases. There was a significant difference in the sensitivities between the Abbott POCT and Architect IgM and IgG results (20%, 95% CI 10.3 to 29.7, p ​= ​0.0002), but not between the Roche POCT and Cobas total antibody results (3.08%, 95% CI -8.18 to 14.3, p ​= ​0.79). There was a significant difference in sensitivity between the Architect and Cobas results (15.4%, 95% CI 2.43 to 28.3, p ​= ​0.04), but not between the Abbott and Roche POCTs (7.69%, 95% CI -0.06 to 15.5, p ​= ​0.13).

### 3.3 Analysis of individual POCT antibody bands

We examined the sensitivity of the individual bands on the Abbott and Roche POCTs. On both the Abbott and Roche POCTs, the sensitivity of the combined IgM and IgG band results were similar to the sensitivity of the IgG band results. The IgM-band on the Abbott POCT was absent in 97.0% (129/133) of cases and did not develop prior to IgG positivity, and on the Roche POCT the IgM-band was absent in 72.9% (97/133) of cases. The Abbott IgM-band had poor sensitivity within the first week of infection (0.00%), which did not improve much even after 2 weeks (PPA 2.78% (95% CI 0.07 to 14.5) ≥14 days POS), with a total sensitivity of 3.01% (95% CI 0.83 to 7.52) (see Supplementary Table 2). The IgM-band on the Roche POCT had a higher sensitivity (7.69% in the first week, 46.9% 7–13 days POS), but also did not increase to >95% after the second week (44.4%) (see Supplementary Table 3).

### 3.4 Predictive values

Assuming a disease prevalence of 5%, when using all controls and cases, the PPV of the Abbott POCT will be 68.7% (95% CI 41.3 to 87.2) [PPV = (67/133)∗0.05/(67/133)∗0.05 ​+ ​[1 – (245/248)]∗(1–0.05)], and the NPV was 97.4% (95% CI 97.0 to 97.8). For the Roche POCT, PPV will be 100.0% (95% CI 94.7 to 100.0) [PPV = (72/133)∗0.05/(72/133)∗0.05 ​+ ​[1 – (248/248)]∗(1–0.05)], and NPV 97.6% (95% CI 97.2 to 98.0).

## 4 Discussion

It is essential that the performance of newly developed POCT assays be thoroughly evaluated before use. Performance between different POCT assays varies greatly (see Supplementary Table 4) [[5], [6], [7], [8], [9], [10], [11], [12]] with a range of different assay sensitivities. The meta-analysis by ML Bastos in July 2020 [3] showed that POCT assays up till April 2020 had varied sensitivities (36.4%–100%). LFIAs since then continue to have sensitivities that can range from 22% to 60.9% [11,12]. Therefore, the recent IFCC guidelines [13] on serological testing for SARS-CoV-2 recommends follow up with a laboratory assay for patients with a high suspicion of infection even when negative POCT results are encountered.

Up to 40–45% of COVID-19 infections may be asymptomatic [14]. SARS-CoV-2 RT-PCR only has a sensitivity of around 79% [15], with a false-negative rate of 38% on the day of disease onset [16]. Indeed, in a study of 164 close contacts of known COVID-19 cases [17], 7 subjects with negative RT-PCR results had positive antibody tests. Thus, the US Centers for Disease Control and Prevention has recommended serologic assays in suspected COVID-19 cases with negative RT-PCR [18]. Antibody responses are uncommon during the first week of infection [19]. However, by 14 days after the initial infection, antibodies are detectable in over 95% of cases [20] and can last up to 4 months later [21].

Both POCTs we evaluated agreed closely with the CLIAs in RT-PCR positive cases and in controls. In cases ≥14 days POS, assay sensitivities were >95% with good agreement (Abbott 97.2% concordance, Roche 100% concordance). This concordance shows that if required, the POCTs can act as a substitute for CLIAs in cases ≥14 days POS. This would be especially useful to centers which do not have access to centralized laboratory facilities. However, caution should be exercised when using POCTs in early infection. The poor sensitivities of the POCTs within the first week of infection (Abbott POCT 10.8%, Roche POCT 18.5%) precludes their use during this period. This is due to seroconversion typically occurring only 14 days after disease onset [22]. Evaluations of several other LFIAs [23] also shows that sensitivity increases appreciably to 91–94% only 14 days after disease onset. CLIAs have the advantage in that they diagnose reactivity based on a reportable COI value. In our previous evaluation of the Abbott Architect IgG assay with a larger study population [1], an initial sensitivity of 45.9% within the first week of infection could be improved to 55.8% by using a lower, optimized COI value for reactivity. Other studies [24] have also suggested that a lower COI value for the diagnosis of reactive samples will improve the sensitivity of CLIAs in their cases with early infection. However, POCTs do not have the ability to report COIs, and thus cannot benefit from this modification to improve sensitivities.

Even after 14 days POS, the Abbott POCT IgM-band was negative in 97.0% (129/133) of cases and the Roche POCT IgM-band was negative in 72.9% (97/133) of cases. These results show that there is little to be gained in using this assay to dissect the individual IgG-IgM components on POCTs for test interpretation. One study that examined the kinetics of SARS-CoV-2 antibodies in COVID-19 infections [25] has shown that IgM does increase in the first week after symptom onset (seropositivity rate of 75%), but this elevation becomes blunted by the second week of infection (IgM seropositivity increasing to 84.2%, IgG to 94.7%) and declines thereafter. A study of 285 patients [17] showed that the seroconversion of IgG and IgM may occur either simultaneously or sequentially. In another study [26] (n ​= ​17,368), IgG responses rose earlier and higher than those of IgM. Liu et al. [27] reported that 40% (n ​= ​32) of patients failed to develop IgM at all. As such, it is imperative that either or both positive IgM and IgG-bands on POCT testing is interpreted as a positive result.

In the review by Bastos et al. [3], the pooled sensitivity of LFIAs was 49.3–79.3%, with a pooled specificity of 94.3–98.2%. Several SARS-CoV-2 antigen tests have been given EUAs by the FDA [28], for example the Abbott BinaxNow (reported sensitivity 97.1%, specificity 98.5%) [29]. A recent Cochrane review of antigen tests showed a pooled sensitivity of 56.2% and specificity of 99.5% [30]. PPVs of LFIAs may be acceptable in an optimized laboratory-based evaluation. However, their performance in the real world will be impaired especially in low prevalence areas. This limitation can be improved when one test is combined with another test (e.g. antigen test) in an orthogonal approach [18]. To investigate this orthogonal effect, we have used the online calculator provided by the FDA to estimate the combined performance of two independent tests [31]. Assuming a population prevalence of 5%, and pooled sensitivities/specificities of the antigen test (sensitivity 56.2%, specificity 99.5%) and LFIAs (sensitivity 49.3–79.3%, specificity 94.3–98.2%) in an orthogonal test format (a positive antigen test followed by an antibody test), the combined PPV would be better than the PPV of each individual test alone (see Table 4).

**Table 4:** PPV of combined use of antigen/antibody tests in orthogonal testing with increasing prevalence.

LFIA sensitivity 49.3%, specificity 94.3%			
Prevalence (%)	Antigen PPV	Antibody PPV	Combined PPV
0.1	10.1	0.9	49.3
0.5	36.1	4.2	83.0
1	53.2	8.0	90.8
5	85.5	31.3	98.1
**LFIA sensitivity 79.3%, specificity 98.2%**			
0.1	10.1	4.2	83.2
0.5	36.1	18.1	96.1
1	53.2	30.8	98.0
5	85.5	69.9	99.6

Abbreviations: LFIA: lateral flow immunoassays, PPV: positive predictive value.

Even if the disease prevalence decreased to 0.1%, the combined PPV would still be superior to the PPV of each individual test alone. This approach would mitigate against false positive results, especially in a low prevalence setting. Some reports [32] have espoused the use of frequent SARS-CoV-2 antigen POCT testing to improve the detection of COVID-19 in the general population. If this is combined with a rapid SARS-CoV-2 antibody test, the false positive rate could be reduced substantially.

The strengths of our study are that we have assessed the cross-reactivity of the assay with commonly found antibody positive cases in other disorders. We have also assessed the sensitivity performance, and concordance with CLIAs, of two newly released POCTs stratified by days POS (0–6/7-13/≥14). Some studies [33] have shown that dengue infections can lead to some false positive cases on other assays. Within our study, dengue antibodies have no cross-reactivity with both POCTs.

We report the following findings for both POCTs:

➢ The IgM-band is negative in most cases of COVID-19.
➢ A negative IgM-band result cannot be used to exclude COVID-19.
➢ Either or both IgG- and IgM-band positivity on the POCT should be considered as a positive result.
➢ There is little utility in examining the IgG-IgM bands individually.
➢ The sensitivity of the POCTs only improves greatly after 14 days POS.
➢ Combining the antibody test with another test (e.g. a SARS-CoV-2 antigen assay) in orthogonal testing can greatly improve the PPV of both assays.
➢ We have provided a compilation of recently evaluated LFIAs for COVID-19 (see Supplementary Table 4) for the benefit of readers.

A limitation of this study is that we only utilized serum samples, without the benefit of whole blood/fingerstick samples more commonly used in POCT situations. Matrix influence can significantly affect POCT results; some studies [11] suggest that for some LFIA assays, serum samples can result in a higher sensitivity than finger prick samples (80% sensitivity vs 22% sensitivity). In all other situations for POCTs, the difference between whole blood/fingerstick versus serum is less than the relative change value. Indeed, since submission we had 2 new cases of COVID-19 in our institution, and on testing with all 3 matrixes there was complete agreement on the POCTs. The population of this study was a hospital population, and in the general population, antibody responses might be lower due to a higher number of asymptomatic/pauci-symptomatic infections [12,34,35]. More severe infections have higher antibody titres than less severe cases. When serial blood samples in 38 patients with severe COVID-19 was compared to 24 mild COVID-19 infections [36], 65% of severe cases demonstrated antibody activity, compared to 30% in mild cases. Liu et al. [27] showed a significant difference in the IgG response between patients with mild and severe infection. In some mild cases (21.43%), an adequate level of IgG antibodies did not develop until 9 days after symptom onset. Weaker antibody responses can cause negative/borderline POCT results. In a study evaluating seven LFIAs [12], patients with lower IgM levels were more prone to produce borderline results on POCT testing; they found 6 asymptomatic RT-PCR positive COVID-19 patients who had repeated negative LFIA results. The sensitivities of the Architect and Cobas CLIAs are also different from our previous evaluations [1,2]. This is because the populations used in the previous studies were larger and had different demographics. Further evaluations of both POCTs using larger populations would be desirable.

## 5 Conclusion

A large proportion of cases do not develop detectable IgM on POCT assays. Individual antibody bands on the POCT provides little additional information. Either or both IgM/IgG-band positivity should be interpreted as reactive. Although both POCT assays have good specificity and little cross-reactivity with other antibodies, their sensitivity only improves to >95% when used in subjects ≥14 days POS; where they can substitute for CLIAs if unavailable.

## Disclosures

All co-authors have contributed to the study and manuscript.

## Declaration of competing interest

The authors declare that they have no known competing financial interests or personal relationships that could have appeared to influence the work reported in this paper.
