# Supplementary material for: Performance of two rapid point of care SARS-COV-2 antibody assays against laboratory-based automated chemiluminescent immunoassays for SARS-COV-2 IG-G, IG-M and total antibodies
Source: Pract Lab Med. 2021 Jan 19;24:e00201. doi: 10.1016/j.plabm.2021.e00201 (PMC7816621; doi:10.1016/j.plabm.2021.e00201)
Supplement: Multimedia component 1 [file mmc1.docx]

**SUPPLEMENTARY TABLES**

**SUPPLEMENTARY TABLE 1: STARD Checklist**

|  | **Section & Topic** | **No** | **Item** | **Reported on page #** |
| --- | --- | --- | --- | --- |
|  |  |  |  |  |
|  | **TITLE OR ABSTRACT** |  |  |  |
|  |  | **1** | Identification as a study of diagnostic accuracy using at least one measure of accuracy (such as sensitivity, specificity, predictive values, or AUC) | **2** |
|  | **ABSTRACT** |  |  |  |
|  |  | **2** | Structured summary of study design, methods, results, and conclusions  (for specific guidance, see STARD for Abstracts) | **2** |
|  | **INTRODUCTION** |  |  |  |
|  |  | **3** | Scientific and clinical background, including the intended use and clinical role of the index test | **4** |
|  |  | **4** | Study objectives and hypotheses | **4** |
|  | **METHODS** |  |  |  |
|  | *Study design* | **5** | Whether data collection was planned before the index test and reference standard  were performed (prospective study) or after (retrospective study) | **5** |
|  | *Participants* | **6** | Eligibility criteria | **5** |
|  |  | **7** | On what basis potentially eligible participants were identified  (such as symptoms, results from previous tests, inclusion in registry) | **5** |
|  |  | **8** | Where and when potentially eligible participants were identified (setting, location and dates) | **5** |
|  |  | **9** | Whether participants formed a consecutive, random or convenience series | **5** |
|  | *Test methods* | **10a** | Index test, in sufficient detail to allow replication | **5** |
|  |  | **10b** | Reference standard, in sufficient detail to allow replication | **5** |
|  |  | **11** | Rationale for choosing the reference standard (if alternatives exist) | **6** |
|  |  | **12a** | Definition of and rationale for test positivity cut-offs or result categories  of the index test, distinguishing pre-specified from exploratory | **6** |
|  |  | **12b** | Definition of and rationale for test positivity cut-offs or result categories  of the reference standard, distinguishing pre-specified from exploratory | **6** |
|  |  | **13a** | Whether clinical information and reference standard results were available  to the performers/readers of the index test | **6** |
|  |  | **13b** | Whether clinical information and index test results were available  to the assessors of the reference standard | **6** |
|  | *Analysis* | **14** | Methods for estimating or comparing measures of diagnostic accuracy | **6** |
|  |  | **15** | How indeterminate index test or reference standard results were handled | **6** |
|  |  | **16** | How missing data on the index test and reference standard were handled | **6** |
|  |  | **17** | Any analyses of variability in diagnostic accuracy, distinguishing pre-specified from exploratory | **6** |
|  |  | **18** | Intended sample size and how it was determined | **Not applicable** |
|  | **RESULTS** |  |  |  |
|  | *Participants* | **19** | Flow of participants, using a diagram | **Not applicable** |
|  |  | **20** | Baseline demographic and clinical characteristics of participants | **5** |
|  |  | **21a** | Distribution of severity of disease in those with the target condition | **Not applicable** |
|  |  | **21b** | Distribution of alternative diagnoses in those without the target condition | **Not applicable** |
|  |  | **22** | Time interval and any clinical interventions between index test and reference standard | **Not applicable** |
|  | *Test results* | **23** | Cross tabulation of the index test results (or their distribution)  by the results of the reference standard | **9** |
|  |  | **24** | Estimates of diagnostic accuracy and their precision (such as 95% confidence intervals) | **8** |
|  |  | **25** | Any adverse events from performing the index test or the reference standard | **Not applicable** |
|  | **DISCUSSION** |  |  |  |
|  |  | **26** | Study limitations, including sources of potential bias, statistical uncertainty, and generalisability | **15** |
|  |  | **27** | Implications for practice, including the intended use and clinical role of the index test | **14** |
|  | **OTHER INFORMATION** |  |  |  |
|  |  | **28** | Registration number and name of registry | **7** |
|  |  | **29** | Where the full study protocol can be accessed | **Not applicable** |
|  |  | **30** | Sources of funding and other support; role of funders | **17** |
|  |  |  |  |  |

**SUPPLEMENTARY TABLE 2: Analysis of the Abbott POCT by individual bands**

| Days POS | N | Positive | Negative | PPA (95% CI) |
| --- | --- | --- | --- | --- |
| IgG band positive | | | | |
| 0 to 6 | 65 | 7 | 58 | 10.8 (4.44 to 20.9) |
| 7 to 13 | 32 | 25 | 7 | 78.1 (60.0 to 90.7) |
| ≥14 | 36 | 35 | 1 | 97.2 (85.47 to 99.9) |
| Total | 133 | 67 | 66 | 50.4 (41.6 to 59.2) |
| IgM band positive | | | | |
| 0 to 6 | 65 | 0 | 65 | 0.0 (0.0 to 5.52) |
| 7 to 13 | 32 | 3 | 29 | 9.38 (1.98 to 25.0) |
| ≥14 | 36 | 1 | 35 | 2.78 (0.07 to 14.5) |
| Total | 133 | 4 | 129 | 3.01 (0.83 to 7.52) |

*Abbreviations: POCT: point-of-care test, POS: post-first positive RT-PCR, PPA: positive percentage agreement.*

**SUPPLEMENTARY TABLE 3: Analysis of the Roche POCT by individual bands**

| Days POS | N | Positive | Negative | PPA (95% CI) |
| --- | --- | --- | --- | --- |
| IgG band positive | | | | |
| 0 to 6 | 65 | 9 | 56 | 13.8 (6.53 to 24.7) |
| 7 to 13 | 32 | 25 | 7 | 78.1 (60.0 to 90.7) |
| ≥14 | 36 | 35 | 1 | 97.2 (85.5 to 99.9) |
| Total | 133 | 69 | 64 | 51.9 (43.1 to 60.6) |
| IgM band positive | | | | |
| 0 to 6 | 65 | 5 | 60 | 7.69 (2.55 to 17.0) |
| 7 to 13 | 32 | 15 | 17 | 46.9 (29.1 to 65.3) |
| ≥14 | 36 | 16 | 20 | 44.4 (27.9 to 61.9) |
| Total | 133 | 36 | 97 | 27.1 (19.7 to 35.5) |

*Abbreviations: POCT: point-of-care test, POS: post-first positive RT-PCR, PPA: positive percentage agreement.*

**SUPPLEMENTARY TABLE 4: A summary of evaluations of current SARS-CoV-2 antibody POCTs**

**Prior to ML Bastos, et al [3]**

| Paper | Study type | Sensitivity | Specificity | Remarks |
| --- | --- | --- | --- | --- |
| YB Pan, et al [5] | Assay evaluation | 1-7 days: 11.1%  8-14 days: 92.9%  ≥15 days: 96.8% | Not reported | Able to detect antibodies in 44.4-71.4% of RT-PCR negative patients |
| M Ricco, et al [6] | Meta-analysis | Overall sensitivity 64.8% | Overall specificity 98% | Meta-analysis of 10 studies that assessed various LFIAs. |
| ML Bastos, et al [3] | Meta-analysis | LFIA tests (POCT): 66% | LFIA tests: 97% | Meta-analysis of 40 studies, included ELISA and CLIA in addition to LFIAs |
| JD Whitman, et al [7] | Meta-analysis | LFIA tests:  1-5 days: 17.9-42.9%  6-10 days: 54.3-77.8%  11-15 days: 73.5-88.2%  16-20 days: 69.2-89.5%  >20 days: 81.8-100% | 84.3-100% | Three pre-COVID specimens were scored positive by more than 3 LFIAs. |
| D Jarrom, et al [8] | Meta-analysis | 18.4 – 88.66% | 88.9 – 100% | Systemic review also included a separate analysis of RT-PCR POCTs. |
| T Prazuck, et al [9] | Assay evaluation | 0-5 days: 10.0%, 35.7%  6-10 days: 58.1%, 54.8%  11-15 days: 69.2%, 81.8%  >15 days: 100%, 100% | 100%, 100% | Two POCT tests, COVID-PRESTO and COVID-DUO were assessed. |

**After ML Bastos, et al [3]**

| Paper | Study type | Sensitivity | Specificity | Remarks |
| --- | --- | --- | --- | --- |
| PP Zhang, et al [10] | Assay evaluation | 86.9% | 99.4% | Laboratory developed assay. |
| B Flower, et al [11] | Assay evaluation | 22-96%  (Sensitivity was found to be lower than manufacturer reported values) | 97.2-99.8% | Concordance between LFIA and laboratory assay: kappa 0.13 to 0.56. |
| S Pickering, et al [12] | Assay evaluation | 60.9-87.3%  (increased detection of antibodies was seen in all tests with increasing time post-symptom onset) | 82-100% | LFIAs were compared to an in-house ELISA |

*Abbreviations: LFIA: lateral flow immunoassay, ELISA: enzyme-linked immunosorbent assays, CLIA:* *chemiluminescent immunoassays, POCT: point-of-care test*
